# Are technical performance differences in wheelchair fencing linked to disability categories?

**DOI:** 10.1186/s13102-025-01071-z

**Published:** 2025-02-18

**Authors:** Zbigniew Borysiuk, Monika Błaszczyszyn, Katarzyna Piechota, Anna Akbaş

**Affiliations:** 1 Faculty of Physical Education and Physiotherapy, Opole University of Technology, Opole, Poland; 2 Institute of Sport Sciences, Academy of Physical Education, Katowice, Poland

**Keywords:** Wheelchair fencers, Surface electromyography, Postural muscles

## Abstract

**Background:**

This study aimed to examine differences in muscle activity and activation timing in wheelchair fencers across disability categories A and B to better understand the neuromuscular dynamics involved in their performance.

**Methods:**

Sixteen right-handed wheelchair fencers from the Polish National Paralympic Team, grouped into categories A and B, participated in the study. Muscle activity and activation timing (reaction time) were recorded during a visual-cue task using a surface electromyography system and a 3D accelerometer. Eight upper body muscles, including the deltoid, triceps, biceps, forearm extensors/flexors, latissimus dorsi, and obliques, were assessed. Data were processed using MyoResearch and MATLAB, and statistical analyses utilized the Wald-Wolfowitz runs test.

**Results:**

Intergroup differences in reaction time and muscle activity were found: category A fencers tended to achieve lower reaction times and higher muscle bioelectric tension values than category B fencers. Significant differences between the groups were found in the activity of the left latissimus dorsi and the deltoid muscles (*p* < 0.05).

**Conclusions:**

The study indicates the important role of the back and abdominal muscles as stabilizing postural muscles in wheelchair fencing. The significant differences in muscle activity for the back and deltoid muscles suggest distinct neuromuscular profiles between disability categories A and B. These findings could enhance classification accuracy and inform training strategies for para-athletes, optimizing performance and targeting specific muscle groups for improvement.

## Introduction

Wheelchair fencing has been part of the Paralympic Games program since the 1960 Rome Games. At the 2006 World Fencing Championships in Turin, the tournaments were held in the integrated format for both able-bodied fencers and fencers with disabilities [1].

The remarkable history of international achievements of disabled fencers makes the sport of wheelchair fencing of great interest to researchers in sport theory, biomechanics, physiology, psychology and physiotherapy [2, 3]. However, literature reviews show a lack of reports on the regulation of motor activities of disabled athletes and within the framework of motor control theory [4]. Current technological advances allow the use of integrated systems for the study of movement, among which the application of electromyography is at the forefront. EMG enables the analysis of the structure of muscle tensions during the performance of technical actions with simultaneous recording of bioelectrical activity times of selected muscles [5]. In addition, different types of perception are studied using high-speed cameras and accelerometers to measure reaction time (RT) and movement time (MT). The use of integrated testing tools provides the opportunity to record the sequence of muscle activation during different technical actions performed by athletes. In this way, an integrated research system provides knowledge of desired movement patterns for the purposes of sports practice and effective coaching, which forms the basis for the development of optimal and effective techniques to be used in sports competition [2, 6].

Another important issue in wheelchair fencing is the use of recording movement patterns according to the degree of disability of the athletes. In wheelchair fencing there are three categories of disability: A, B, C [7]. Category A includes the relatively fittest fencers, such as amputees or individuals with mild paralysis of the lower limbs; Category B includes fencers with spinal cord injuries and paraplegia; and Category C includes individuals with tetraplegia. The present study included fencers representing categories A and B. Due to the fact that the majority of participants competed in all three fencing disciplines (foil, epee, saber) at the same time, it was not necessary to divide them into three separate weapon categories [8].

Wheelchair fencing mirrors fencing for able-bodied athletes in terms of techniques, strategy, and scoring systems. While the refereeing, scoring, and competition rules are similar, wheelchair fencers face unique challenges due to their fixed position in the chair, which requires greater emphasis on upper-body coordination and trunk stabilization. In able-bodied fencing, the legs play a significant role in mobility [9, 10], but wheelchair fencers rely more on their upper bodies to maintain balance and control, especially the shoulders and forearms, as well as the trunk (back and abdomen) [8, 11]. Training in both sports involves individual lessons and sparring to prepare for tournaments, but wheelchair fencers must adapt their techniques to their stationary position.

Both traditional and wheelchair fencing are psychomotor sports that require a combination of coordination skills (reaction time, speed, proprioception, attention) and performance abilities (endurance, explosiveness) [12, 13]. However, the physical constraints of wheelchair fencing make reaction time and speed even more critical, as athletes must compensate for their lack of mobility by focusing on maintaining balance and controlling the chair, which directly impacts performance. Attack maneuvers are crucial, with priority given to the attacking fencer, similar to able-bodied fencing [14]. Physiological and anthropometric factors influence speed of attack; in wheelchair fencing, a higher sitting height and greater arm span improve reach and speed, compensating for the lack of leg movement [4].

The design of fencing wheelchairs allows for extraordinary dynamics of torso and sword arm movements, which determine the fencer’s motor skills. In addition, the fencers are strapped into the wheelchairs to prevent them from falling out. The design of fencing wheelchairs enables exceptional dynamics in torso and sword arm movements, which are crucial for the fencer’s motor performance. To ensure safety, fencers are securely strapped into the wheelchair, which is anchored to a specialized frame. The wheelchair must meet specific regulations: it must remain flexible, with a maximum seat height of 53 cm, a backrest at least 15 cm high positioned at a 90º angle (with certain exceptions), and an armrest on the non-dominant side measuring at least 10 cm. While not mandatory, cushions are permitted with a maximum height of 10 cm. Additionally, the angle of the wheelchair’s wheels must adhere to official specifications [15].

Numerous studies of able-bodied fencers have demonstrated the role of the legs, which perform important postural functions, in structuring movement patterns [9, 16]. For wheelchair fencers, the sword-arm muscles, along with key abdominal and trunk muscles like the external abdominal oblique and latissimus dorsi, play a vital role in maintaining postural balance [17, 18].

The main aim of the study was to identify neuromuscular determinants of EMG recorded movement patterns in wheelchair fencers. In addition, the reaction time (RT) of fencers’ technical actions to the coach’s signal was determined using an accelerometer. The differences in muscle activity and the speed of muscle activation between fencers representing disability categories A and B were then analyzed. The results of the study can contribute to the development of more precise criteria for the classification of disabled fencers into disability categories A and B.

## Materials and methods

### Participants

The study involved 16 wheelchair fencers from the Polish National Paralympic Team, classified into disability categories A and B. The purpose of comparing category A and category B fencers was to explore how varying levels of muscle activation (resulting from differences in impairments) affect performance in fencing. Category A athletes, who generally experience less severe impairments, are able to activate muscles involved in fencing more fully and may demonstrate muscle patterns more typical of able-bodied athletes. On the other hand, category B athletes often have limited or altered muscle activation due to neurological or musculoskeletal conditions, which may lead to adaptive strategies in how they control their weapons and execute movements. Detailed characteristics of each group are provided in Table 1. All participants were right-handed. The study adhered to ethical standards as outlined in the Declaration of Helsinki for clinical research involving human subjects and was approved by the Bioethics Committee of the Chamber of Physicians (Resolution No. 237, December 13, 2016). Prior to participation, all individuals were fully informed about the study procedures and provided written informed consent.

**Table 1:** Demographical and clinical features of wheelchairs fencers in categories a and B

Variables		Groups	
		Category A	Category B
*N*		7	9
Age (years), mean ± SD		32.57 ± 6.25	30.33 ± 9.67
Height (m), mean ± SD		1.74 ± 0.09	1.64 ± 0.11
Mass (kg), mean ± SD		69 ± 8.93	58.67 ± 10.3
Sex, *n*			
M		3	5
F		4	4
Training experience (years), mean ± SD		14.29 ± 6.32	6 ± 2.4
Disease entity, *n*			
amputation		2	˗
stroke		1	˗
paraplegia		1	4
musculoskeletal amputation		1	˗
cerebral palsy		1	˗
myelomeningocele		1	˗
multiple sclerosis		˗	1
hernia		˗	1
spina bifida		˗	1
post-operative lower limb paralysis		˗	1
lower limb palsy		˗	1

Legend: M– male, F - female

### Apparatus

**Fig. 1:**
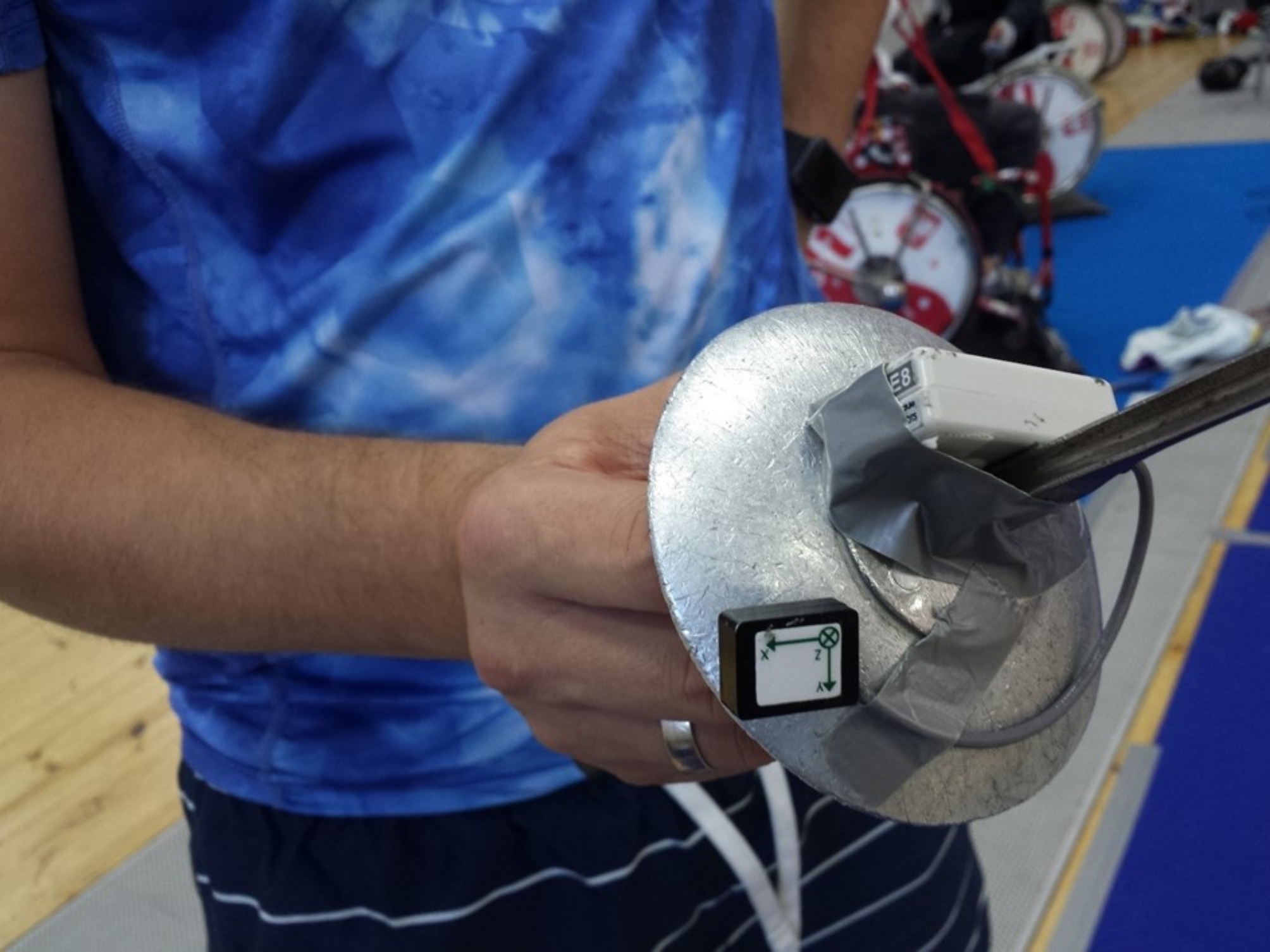
Preparation of the trainer’s weapon with attached accelerometer

A 16-channel surface electromyography (sEMG) system (Noraxon, DTS, Desktop Direct Transmission System, Scottsdale, Arizona, USA) with a sampling frequency of 1500 Hz and 16-bit resolution was used to record the electrical activity of the muscles. A 3-axis wireless DTS 3D accelerometer sensor (± 6 g nominal output range, ± 0.67 V/g sensitivity, 5 Hz − 1.8 kHz bandwidth) was attached to the guard of the trainer’s epee to detect the onset of the stimulus (Fig. 1). Data analysis was conducted using dedicated software (MyoResearch XP Master Edition for DTS Noraxon).

A wireless transmitter-recorder was employed to synchronize the EMG system and wirelessly transmit the EMG signals directly to the PC. To minimize impedance skin preparation followed standard procedures, including cleaning the skin over the relevant muscle groups. EMG electrodes were placed on the muscle bellies, perpendicular to the muscle fibers, following SENIAM guidelines. Electrodes were applied unilaterally on the right arm to the forearm muscles (extensor carpi radialis longus (ECR) and flexor carpi radialis (FCR)), the arm muscles (deltoid middle head (DEL), triceps brachii (TRI), and biceps brachii (BC), and bilaterally on the trunk muscles (external abdominal oblique (EAO) and latissimus dorsi (LT).

### Procedures

The experimental procedures were adapted from the methodology described by Błaszczyszyn et al. [19]. Each session began with a warm-up, during which athletes participated in a 20- to 25-minute individualized training session with their coach. The warm-up consisted of a combination of general and sport-specific exercises tailored to the athletes’ habitual training routines. General exercises included movements to activate major muscle groups, improve joint mobility, and increase overall blood flow, while sport-specific drills focused on refining fencing techniques, reaction times, and coordination. Following the warm-up, participants were prepared for sEMG measurements.

The athletes were then positioned for testing. Each fencer sat in their wheelchair, which was securely mounted on a platform to ensure stability. The athletes were then positioned for testing. Each fencer sat in their wheelchair, which was securely mounted on a platform to ensure stability. The fencer’s feet were placed flat on the footrests, adjusted to the appropriate height for each individual. The hips were positioned at a 90-degree angle, with the back kept straight. The arms were held comfortably, with slightly bent elbows, while the hands and wrists remained relaxed. The wheelchair was positioned as close to the piste as possible, with the weapon arm slightly extended forward. The use of the non-weapon arm was prohibited according to the regulations [15]. During a lunge, the wheelchair was pushed forward with the weapon arm fully extended, while the rear foot remained in contact with the floor. When retreating, the wheelchair was pushed backward with the weapon arm held in a stable position. Maintaining control of the wheelchair was critical during both lunges and retreats [15].

**Fig. 2:**
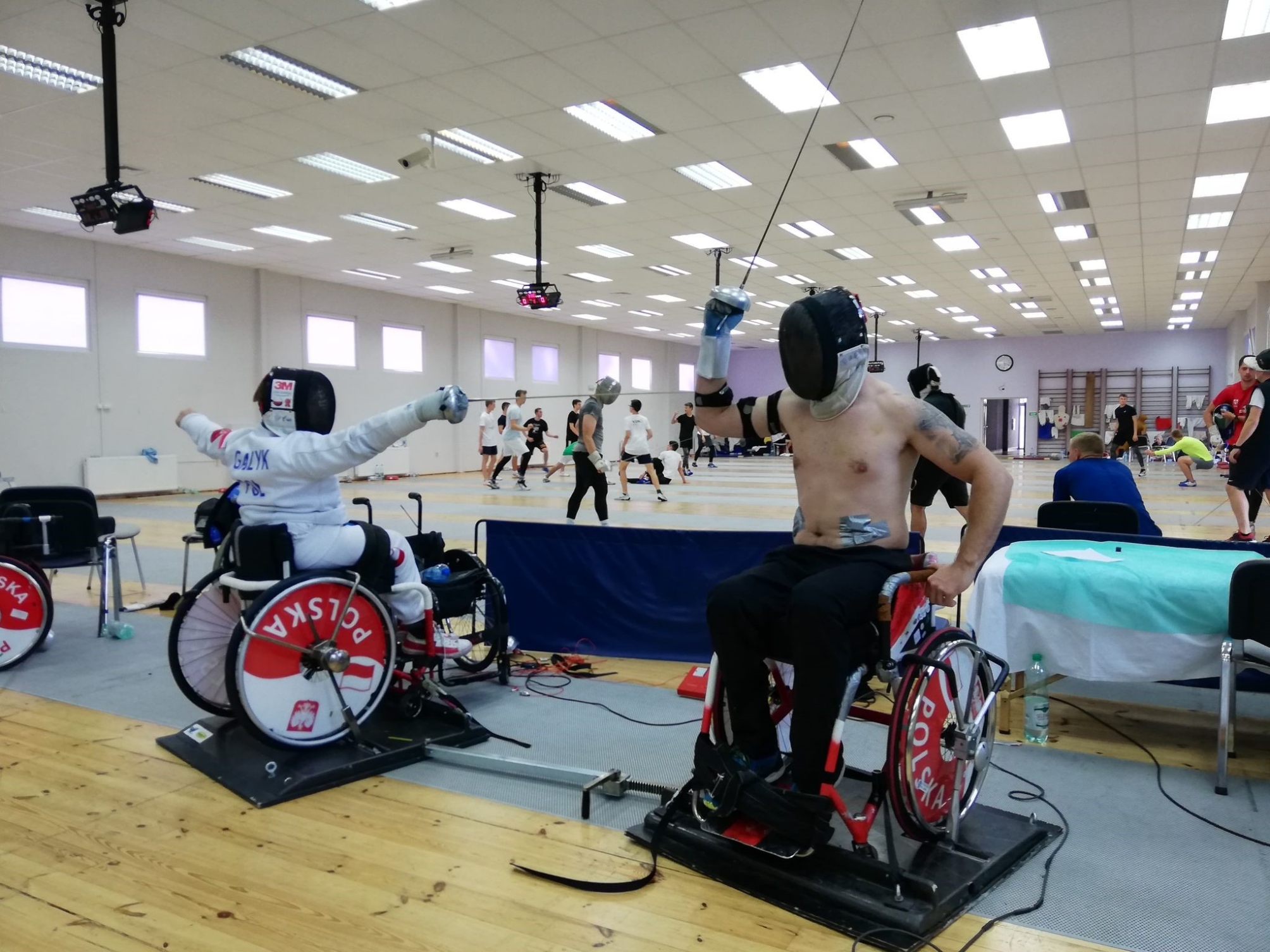
Experimental Setup: Proper distance alignment for fencers during testing

The setup maintained a consistent distance between the tip of the athlete’s weapon and the trainer’s bent elbow (Fig. 2). Both the athlete and trainer sat side-by-side, with their dominant sides facing each other, while the athlete’s non-dominant upper limb was immobilized on the wheelchair for standardization.

The task involved a reaction to a visual cue. After assuming the starting position and hearing the “ready” command, the athlete waited for the trainer to give a visual signal– a quick movement of the trainer’s weapon. Upon seeing the signal, the athlete performed a thrust as quickly as possible, followed by a return to the starting position. This procedure was repeated three times to ensure consistency and reliability of the data.

### Data analysis

The sEMG signals were smoothed by estimating the square root mean within 100 ms. One of the EMG indicators was the determination of a maximum value using the MyoResearch XP Mater Edition software. The maximum EMG value was obtained after data normalization of three trials. The reference value for the maximal voluntary contraction (MVC) was calculated in a time window of 50 ms in which the mean value of the sEMG signal was highest. All signals were normalized to these values and expressed in percent. Of the three tests that were carried out, the second test was the most frequently subjected to a detailed analysis.

The sequence of bioelectric muscle activation and thus the reaction time of the athletes was determined based on a baseline determined by the peak values of the selected muscle groups. MyoResearch XP Mater Edition and Matlab (version 2018b) software was used to determine the baseline threshold to determine the moment corresponding to the start and end of muscle activation (Onset/Offset). The method used to estimate the Onset and Offset thresholds was to determine the local peak value = 5%.

### Statistical analysis

The collected data were processed using Statistica 13.1 (StatSoft, Inc., Oklahoma, USA). The Shapiro-Wilk test was used to verify the assumption of normal distribution of the analyzed statistics. The main research assumption regarding the differences between the groups was tested with the non-parametric Wald-Wolfowitz runs test. Hypotheses were tested at the level of statistical significance of *p* ≤ 0.05.

## Results

**Fig. 3:**
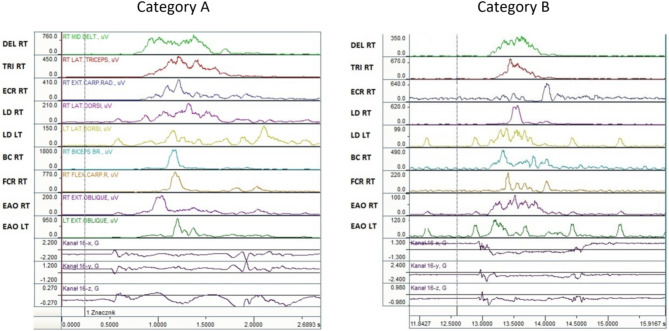
Muscle activation sequences in a representative wheelchair fencers across different disability categories

To better illustrate the activation patterns, Fig. 3 presents the order of activation of nine muscles in a representative wheelchair fencer from disability category A (left panel) and category B (right panel).

For disability category A, the sequence of muscle activation reaction times (RT) was as follows: the dorsal muscles LD RT and LD LT (right and left side), responsible for the anticipatory forward lean of the trunk, were activated first. Next, the DEL muscle was activated as an arm extensor, followed by the right abdominal muscle, which was synchronized with the sword arm. Subsequently, the ECR and TRI muscles were activated as extensors. The sequence concluded with the activation of the flexor muscle of the sword arm (BC RT) and the left abdominal muscle of the passive arm (EDA LT).

In category B fencers, a different sequence of activation reaction times was observed. First, the forearm muscle (ECR RT) was activated, followed by the abdominal muscles (EDA LT and EDA RT) and the dorsal muscles (LD LT and LD RT) as postural stabilizers. Next, the arm muscles (BC RT, DEL RT, and TRI RT) were activated as extensors. The sequence concluded with the activation of the forearm flexor muscle (FCR RT).

Table 2 presents the means and statistical differences in RT for selected muscles between disability groups A and B.

**Table 2:** Wald-Wolfowitz runs test of mean reaction time (RT) for selected muscles in category A and B fencers

Muscle	Category A (ms)	Category B (ms)	Z	*p*-value
DEL RT	0.490	0.542	1.324	0.184
TRI RT	0.562	0.584	0.492	0.632
ECR RT	0.384	0.529	1.341	0.186
LD RT	0.426	0.572	-0.372	0.728
LD LT	0.334	0.529	-2.064	0.041*
BC RT	0.540	0.522	0.488	0.631
FCR RT	0.622	0.638	0.496	0.638
EAO RT	0.412	0.493	-0.372	0.718
EAO LT	0.437	0.460	0.488	0.640

The study revealed a general tendency for shorter RT in muscle activation among fencers in category A. Notably, a statistically significant difference was observed only for the left latissimus dorsi muscle (LD LT) (*p* = 0.041) (Table 2).

Table 3 presents the statistical differences in EMG activity, expressed as a percentage of maximal voluntary contraction (%MVC), between fencers from disability groups A and B.

**Table 3:** Wald-Wolfowitz runs test of mean EMG activity (%MVC) for selected muscles in category A and B fencers

Muscle	Category A (%MVC)	Category B (%MVC)	Z	*p*-value
DEL RT	115.78	67.60	-2.064	0.041*
TRI RT	72.90	44.98	1.342	0.184
ECR RT	64.12	73.90	-1.219	0.231
LD RT	96.37	48.68	-1.216	0.229
LD LT	97.14	59.23	-0.368	0.722
BC RT	49.87	38.59	0.426	0.634
FCR RT	79.62	62.20	-0.366	0.719
EAO RT	78.43	44.83	-0.374	0.724
EAO LT	89.14	96.84	1.338	0.186

The results indicate a general trend toward higher %MVC values in category A fencers for most of the muscles analyzed. However, a statistically significant difference was observed only for the DEL RT muscle (*p* = 0.041) (Table 3).

## Discussion

The study design was based on the assumption that a differentiating factor between wheelchair fencers in disability categories A and B would be the muscle tension structure determined by the order of activation of key muscles during the execution of a fencing thrust on the coach’s torso [8]. The analysis of the reaction time of the activated muscles was performed and the criteria of movement quality were evaluated by recording the EMG signal values expressed in percent of maximal voluntary contraction (% MVC).

Fencers in group A began the movement sequence by activating their back muscles. Then the DEL was activated, followed by the abdominal muscles (EAO). Later, the activities of the forearm muscles (ECR RT) and the extensor muscles (TRI RT) were recorded. At the end of the thrust, the flexor muscles (BC RT and FCR RT) were activated. In group B fencers, the muscle activation sequence started with the deltoid and then the abdominal muscles. The rest of the sequence was similar to that of group A fencers.

The importance of the trunk and abdominal muscles that perform postural functions in wheelchair fencers should be emphasized, as it is a common denominator for both categories of athletes with disabilities. These muscles are activated first or in synergy with the shoulder extensors. A high dynamic of movement leads to an imbalance, and in order to maintain a stable posture, the central nervous system triggers an anticipatory mechanism that activates the work of the postural muscles first.

Considering the mechanisms of reaction/activation speed of the studied muscles, the reaction times of the category A fencers were shorter than those of the category B fencers. The fastest reaction was recorded for the dorsal muscles and, interestingly, for the left dorsal muscle, which was activated counter laterally to the muscles of the sword arm.

When comparing the bioelectrical tensions of the studied muscles between the groups, it can be seen that the fencers of group A are superior to the fencers of group B in terms of earlier activation of most of the muscles. Group A fencers produce higher EMG signal values expressed in %MVC. This is especially true for the DEL RT shoulder muscle of the sword arm. Therefore, the interpretation that the smaller motor deficits of group A fencers allow for greater mobilization of motor units, as expressed by EMG, is justified.

It can be concluded that the degree of disability determines the level of motor skills of wheelchair fencers, resulting from differences in proprioceptive neuromuscular facilitation. The authors’ study of upper limb motor patterns in paraplegics showed the presence of motor disorders in both limbs, i.e. also in the non-paraplegic limb. These motor disorders were observed in the smoothness and coordination of movements [19, 20]. Based on this, neuromuscular transmission is significantly altered in fencers of both disability categories (A and B), even in uninjured body segments. Therefore, the skills acquired and developed by wheelchair fencers depend on the individual functional status of the athletes, including neuromuscular dysfunction [21]. The explanation for these differences and the specificity of the movement patterns of wheelchair fencers should be sought in the mechanisms of two complementary phenomena: compensation and anticipation. The compensatory system quickly adjusts postural imbalances in wheelchair fencers by activating synergistic muscles. The anticipatory postural adjustment (APA) phenomenon plays an important role as well [22]. Thanks to it, wheelchair fencers can move dynamically from leaning the trunk forward during attacks and backward during defensive actions by using the postural muscles [23].

The differences in movement patterns between wheelchair fencers in disability categories A and B in the present study show that athletes representing lower levels of dysfunction respond more quickly to visual stimuli and generate higher EMG signal values, recruiting more motor units into their muscle activities. As the study showed, the structure of the movement patterns of the two groups of wheelchair fencers is not identical. The above observations show that possible competitions between athletes with different disabilities do not meet the criterion of equality of opportunity. In this sense, the research approach presented in this article and the use of motor control research tools can be used to more accurately classify para-athletes into specific disability categories, not only based on medical criteria [24, 25].

### Limitations

This study has several limitations that should be considered in future research. One of the key factors is the variability in seat height and arm span among participants, which may influence performance outcomes. Additionally, the small sample size, stemming from the low participation rates in wheelchair fencing and the challenges of recruiting elite athletes, limits the generalizability of the findings. Another limitation is the assessment of functional status, which, despite being aligned with assigned sport classifications, may not fully capture the differences in athletes’ capabilities.

## Data Availability

Data are available from the first author upon request.

## Abbreviations

MT: Movement time
RT: Reaction time
sEMG: Surface electromyography
MVC: Maximal voluntary contraction
RT: Right
LT: Left
ECR: Extensor carpi radialis longus
FCR: Flexor carpi radialis
DEL: Deltoid middle head
TRI: Triceps brachii
BC: Biceps brachii
EAO: External abdominal oblique
LT: Latissimus dorsi
APA: Anticipatory postural adjustments
